# An Investigation into Occasional White Spot Syndrome Virus Outbreak in Traditional Paddy Cum Prawn Fields in India

**DOI:** 10.1100/2012/340830

**Published:** 2012-04-19

**Authors:** Deborah Gnana Selvam, K. M. Mujeeb Rahiman, A. A. Mohamed Hatha

**Affiliations:** Department of Marine Biology, Microbiology, and Biochemistry, Cochin University of Science and Technology, Lakeside Campus, Cochin 682 016, India

## Abstract

A yearlong (September 2009–August 2010) study was undertaken to find out possible reasons for occasional occurrence of White Spot Syndrome Virus (WSSV) outbreak in the traditional prawn farms adjoining Cochin backwaters. Physicochemical and bacteriological parameters of water and sediment from feeder canal and four shrimp farms were monitored on a fortnightly basis. The physicochemical parameters showed variation during the two production cycles and between the farms studied. Dissolved oxygen (DO) content of water from feeder canal showed low oxygen levels (as low as 0.8 mg/L) throughout the study period. There was no disease outbreak in the perennial ponds. Poor water exchange coupled with nutrient loading from adjacent houses resulted in phytoplankton bloom in shallow seasonal ponds which led to hypoxic conditions in early morning and supersaturation of DO in the afternoon besides considerably high alkaline pH. Ammonia levels were found to be very high in these ponds. WSSV outbreak was encountered twice during the study leading to mass mortalities in the seasonal ponds. The hypoxia and high ammonia content in water and abrupt fluctuations in temperature, salinity and pH might lead to considerable stress in the shrimps triggering WSSV infection in these traditional ponds.

## 1. Introduction

Across the globe, aquaculture industry is one of the rapidly growing industries in food sector. Aquaculture production in India alone has increased by 72% (2.1 million metric ton) within a decade from 1990 to 2000, compared to the 150% increase in production worldwide. Shrimp production trend in India has shown a steady rise from 1990 to 2003 [1]. Shrimp production from coastal aquaculture during 2004 stood at approximately 120,000 tonnes. Farmed shrimp accounts for about 60% of shrimp exported from the country [2]. The penaeid shrimps have tend to dominate brackish-water culture due to high-value, short production cycles and accessible technologies. Production has increased almost exponentially since the mid-1970s and now accounts for about 58 per cent of aquaculture production from brackish water (72% by value) [3].

The sustainability and development of shrimp aquaculture are largely at stake, as significant ecological and pathological problems are increasing in vast majority of the shrimp producing countries. The production is regularly and seriously affected by problems linked to environmental degradation and to infectious and noninfectious diseases [4]. Of the infectious diseases, bacterial and viral infections, either as single or multiple pathogen conditions, caused most of the production losses. White spot syndrome virus (WSSV) first appeared as an epidemic in penaeid shrimp farms in 1993 in China and then quickly spread in Asian countries and subsequently to all over the world [5]. WSSV has resulted in high mortality in many cultured penaeid shrimp species and huge shrimp production losses. White spot syndrome virus (WSSV) has been reported to cause severe mortalities of cultured penaeid shrimp in several parts of Asia including India [6–8]. There is still no efficient measure to control the disease [9] and the loss caused by this virus has been estimated to be several million dollars in different parts of India. The most surprising feature of this virus is its wide range of potential hosts such as several species of penaeid shrimp, a wide range of other decapods including crabs and more distantly related crustaceans such as the freshwater prawn, *Macrobrachium rosenbergii*. Crabs and planktonic shrimp species which are common inhabitants of shrimp ponds can serve as asymptomatic carriers of WSSV, thus posing a serious threat to the shrimp farming industry. It has been suggested that copepods and perhaps even aquatic insect larvae may also be infected [10]. In India, WSSV has been reported in noncultured crustaceans of shrimp farms like Pest shrimp, *Acetes* sp., *Macrobrachium rosenbergii*, Mud crab *Scylla serrata*, Pest crab *Sesarma oceanica*, and *Pseudograpsus intermedius* [11].

Tiger prawn, *Penaeus monodon* and Indian white prawn *Fenneropenaeus indicus* are the major cultivated species in the paddy cum prawn culture system along Cochin backwaters. Paddy cum prawn culture is a modified farm with occasional inputs of seeds and without any control of water quality parameters. Disease outbreaks are intermittent which are mostly looked at from viral outbreak point, especially WSSV. Though the Cochin backwaters and the feeder canal linked to them have undergone considerable change over the years, no attempt has been made to study the key water quality parameters that are important to the survival of shrimps. Stressors, which are usually related to the phyisiochemical properties of both water and pond bottom which compromise the shrimp immune system thus increasing the rate of WSSV infection [12].

The objective of the study was to identify possible environmental trigger for WSSV outbreak in the paddy cum prawn fields along Cochin backwaters by regular monitoring of the water and sediment quality parameters of the feeder canal and couple of seasonal and perennial ponds.

## 2. Materials and Methods

### 2.1. Study Area and Shrimp Farming Practices

Sampling site is located along Cochin backwaters at Edavanakkad, Cochin, Kerala. Shrimp farming in this region is generally a kind of extensive aquaculture, which is carried out in paddy fields adjoining the Cochin backwaters. This type of shrimp farming is known as paddy-cum-prawn culture and usually carried out soon after the harvesting of the paddy. The decaying paddy straw used to provide enough detritus food for the prawn larvae which used to enter the paddy fields during high tide. The larvae are retained in these ponds for 4-5 months, during which no controls in environmental parameters are exerted. Tidal amplitude between high and low tide is used to partially drain the pond water through a sluice gate, where net is placed and prawns are caught.

For the present study two types of farms were selected: seasonal and perennial ponds of different sizes and depths. The farms in this area are well connected to the Feeder canal system that brings water to this area from Cochin backwater. Exchange of water is carried out in the farms through the sluice gate according to the tidal fluctuation. For the present study, water, sediment, and shrimp samples were collected from Feeder canal, 2 seasonal ponds, and 2 perennial ponds. 

### 2.2. Collection of Samples

Water and sediment samples were collected on a fortnightly basis for water quality parameters, while for bacteriological analysis, samples were collected on a monthly basis. For analysis, water samples were collected in sterile bottles from a depth of 0.1 to 0.5 meter from the surface and sediment samples were collected with mud sampler and transferred to sterile jars. Water and sediment samples were collected from different locations of each sampling site and the samples were pooled before analysis.

### 2.3. Analysis of the Physicochemical Parameters

Temperature, salinity, and pH of the water were measured *in situ* using centigrade thermometer, salinity refractometer (Atago, Japan), and hand-held digital pH meter (Eutech, Singapore), respectively. Temperature and pH of sediment were also measured *in situ* using centigrade thermometer and hand-held digital pH meter (Eutech, Singapore). The dissolved oxygen, alkalinity, total hardness, total ammonia nitrogen, nitrite, and inorganic phosphate of water samples were estimated as per APHA (1998) method. Nitrate was estimated by Resorcinol method [13].

### 2.4. Bacteriological Analysis

Samples for bacteriological analysis were kept in icebox soon after collection and brought to the laboratory. Analysis was completed within 4 hr of collection. Water samples were serially diluted up to 10^−3^ and sediment samples up to 10^−5^ using sterile distilled water. For the analysis of shrimp samples, they were weighed, homogenized using sterile mortar and pestle, and serially diluted to 10^−6^. Aseptic procedures were strictly followed during processing of samples.

### 2.5. Estimation of Total Viable Count

Aliquots of 0.1 mL samples from each dilution were spread plated in triplicate on tryptone soya agar (TSA) or 1/2 strength Zobell's marine agar (1/2 ZMA) or ZMA depending on the salinity of the water samples for the enumeration of total aerobic heterotrophic bacteria, which is expressed as total viable count (TVC). The plates were then incubated at 30°C for 48 h. After incubation, plates with 30 to 300 colonies were selected for counting and isolation of bacteria.

### 2.6. Estimation of Total Coliform

Aliquots of 0.1 mL sample from each dilution were spread plated in triplicate on Mac Conkey agar for the enumeration of total coliform (TC). The plates were incubated at 37°C for 48 h. After incubation, plates were selected for counting, typical coliform like colonies were counted and expressed as TC.

### 2.7. Estimation of Total Vibrio Count

Aliquots of 0.1 mL samples from each dilution were spread plated in triplicate on thiosulphate citrate bile sucrose (TCBS) agar for the enumeration of *Vibrio *like organism, which is expressed as total *Vibrio* like organism (TVLO). The plates were incubated at 30°C for 48 h. After incubation, plates were selected for counting, typical *Vibrio* like colonies were counted and expressed as TVLO.

### 2.8. Estimation of Total Aeromonas Count

Aliquots of 0.1 mL samples from each dilution were spread plated in triplicate on starch ampicillin (SA) agar for the enumeration of* Aeromonas *like organism, which is expressed as total *Aeromonas* like organism (TALO). The plates were incubated at 30°C for 48 h. After incubation, plates were selected for counting. Typical *Aeromonas* like colonies were counted and expressed as TALO.

### 2.9. Isolation and Identification of Total Heterotrophic Bacterial Isolates

After recording the morphological characters and pigmentation, representative types constituting at least 20–40 numbers of colonies were selected from each plate and restreaked onto TSA, 1/2 ZMA or ZMA plates to ensure purity. All the purified isolates were maintained on TSA or 1/2 ZMA or ZMA slants for further characterisation and identified to generic level using the taxonomic key for identification by [14–17]. The various tests carried out included Gram staining, spore staining, motility test, Kovac's oxidase test, oxidation fermentation test, catalase test, acid production in glucose, and Voges-Proskauer test.

### 2.10. Isolation and Identification of Vibrio Isolates

*Vibrio* colonies were isolated according to the colouration of the colonies in TCBS plates. The isolated colonies were restreaked onto TSA plates to ensure purity. All the purified isolates were maintained on TSA slants for further characterisation and identified up to species level using the following biochemical test as per U.S. Food and Drug Administration's Bacteriological Analytical manual (USFDA BAM). The following tests were used to characterise the vibrios: oxidase test, arginine dihydrolase test, ornithine decarboxylase, lysine decarboxylase test, growth in 0%, 3%, 6%, 8%, and 10% NaCl, growth at 42°C, acid from Sucrose, D-cellobiose, lactose, arabinose, D-mannose, and D-mannitol, ONPG (Ortho Nitrophenyl-**β**-galactoside), test and Voges-Proskauer test.

### 2.11. Isolation and Identification of Aeromonas Isolates

After recording the starch utilisation capacity on SA agar, colonies were selected from plate and restreaked onto TSA plates to ensure purity. All the purified isolates were maintained on TSA slants for further characterisation and identified to species level using the following biochemical test as per analytical profile index 20E (API 20E). The tests used were ONPG (orthonitrophenyl-*β*-galactoside) test, arginine dihydrolase test, lysine decarboxylase test, ornithine decarboxylase test, citrate utilization test, H_2_S production test, urease test, tryptophan deaminase (TDA) test, indole production test, Voges Proskauer test, gelatinase test, and acid from glucose, mannitol, inositol, sorbitol, rhamnose, sucrose, amygdalin, melibiose, arabinose, and oxidase production.

### 2.12. Detection of White Spot Syndrome Virus (WSSV) from Shrimp Samples

The deproteinised genomic DNA of the shrimp was prepared according to the method for preparation of genomic DNA from mammalian tissue [18]. Briefly, 200 mg muscle tissue excised from the abdomen of the shrimp was rapidly frozen and crushed to a fine powder. The processed tissue was placed in 2.4 mL digestion buffer (100 mM NaC1, 10 mM Tris-HC1, pH 8, 25 mM EDTA, pH 8, 0.5% sodium dodecyl sulfate, 0.1 mg/mL proteinase K) and incubated at 65°C for 12 to 18 h. The digest was deproteinised by successive phenol/chloroform/isoamyl alcohol extractions, recovered by ethanol precipitation, and dried and resuspended in 0.1 X TE buffer at 65°C for 30 min, and then stored at 4°C until use for PCR.

Oligonucleotide primers (146F and 146R) were used for the amplification of WSBV DNA fragments. Primers 146F and 146R were designed on the basis of the DNA sequence of a cloned WSBV 1461-bp *Sal* I DNA fragment in recombinant plasmid (pms146). The following are the sequences of the primers: 146F1, 5′-ACT ACT AAC TTC AGC CTA TCT AG-3′; 146R1, 5′-TAA TGC GGG TGT AAT GTT CTT ACG A-3′. With this primer set, a 1447-bp fragment is expected to be amplified from WSBV genomic DNA. The internal primers (146F2, 5′-GTA ACT GCC CCT TCC ATC TCC A-3′; and 146R2, 5′-TAC GGC AGC TGC TGC ACC TTG T-3′) were used to confirm that the amplified fragment was indeed from the WSBV 941-bp *Sal *I DNA fragment. The deproteinised DNA samples used for amplification totaled 0.1 to 0.3 pg in a 100 *μ*L reaction mixture containing 10 mM Tris-HC1, pH 9 at 25°C, 50 mM KC1, 1.5 mM MgC1_2_, 0.1% Triton X-100, 200 *μ*M each of dNTP, 100 pmol each of primer, 2.5 units of Taq DNA polymerase. The amplification was performed in a thermal station for 1 cycle of 94°C for 4 min, 55°C for 1 min, 72°C for 3 min; and then 39 cycles of 94°C for 1 min, 55°C for l min, 72°C for 3 min; plus a final 5 min extension at 72°C after 40 cycles. The PCR products were analyzed in 1% agarose gel containing ethidium bromide at a concentration of 0.5 *μ*g/mL and visualized under ultraviolet transillumination [19].

## 3. Results

### 3.1. Water Quality Parameters

The temperature of water remained within the acceptable limit required for prawn culturing for most of the culture period. The minimum temperature encountered was 27°C (July, 2010) and it reached up to 37°C (April, 2010) without any further increase throughout the study period. The sediment temperature was always found to be equal or one or two degrees lesser than that of water (Tables 1 and 2). pH of the sediment ranged between 6.38 to 8.36 which falls within the limit that is necessary for the development of shrimps. The mean values of physicochemical parameters of water recorded during the study period are given in Table 1. Though the pH of water was normal in all the ponds, an alkaline pH of 9.05 (which coincided with heavy algal growth) was encountered in seasonal pond-2 at the start of the culture period (October, 2009). But as the culturing progressed, it gradually decreased and reached a normal level. There was a sharp increase in pH (9.7) again during the monsoon season (July, 2010) and remained high for the rest of the study period. The salinity of water was very low in September (0 ppt) which gradually increased and reached a maximum of 30.8 ppt in February. It is attributed to the variations in the salinity of the water from monsoon to the postmonsoon season. Though the salinity was low initially, good growth of the shrimps could be observed, and there was no mortality during that period.

Dissolved oxygen levels were relatively low in the feeder canal (as low as 0.8 mg/L), and in the case of the other ponds, they were within the levels conducive for shrimp aquaculture. In seasonal pond-2, the dissolved oxygen levels showed much fluctuations (4.5 to 13.8 mg/L) and alkaline pH (9.7) which coincided with algal blooms. Thus, seasonal hypoxic conditions were observed in this pond during our study. Total hardness level was found to be between 300–4400 mg CaCO_3 _mg/L. It was found to increase with increasing salinity and reached a maximum during the summer season and declining as the monsoon started. 

The levels of ammonia were higher than the acceptable limit (0.01 mg/L) for penaeid shrimps. The levels are very high in the month of November (perennial pond-2 recording 1.87 mg/L) and towards the end, the levels are slightly higher. In the shrimp ponds and the feeder canal, TAN values were between 0.0039–1.87 mg/L. The nitrite levels recorded during the study are very low and were well below the safe level (1.28 mg/L) of nitrite recommended for* Penaeus monodon* by Law [20]. Phosphate and nitrate values recorded were between 0.01–0.87 mg/L and 0.005–0.428 mg/L, respectively.

### 3.2. Bacteriology of the Ponds and Feeder Canal

**Figure 1:**
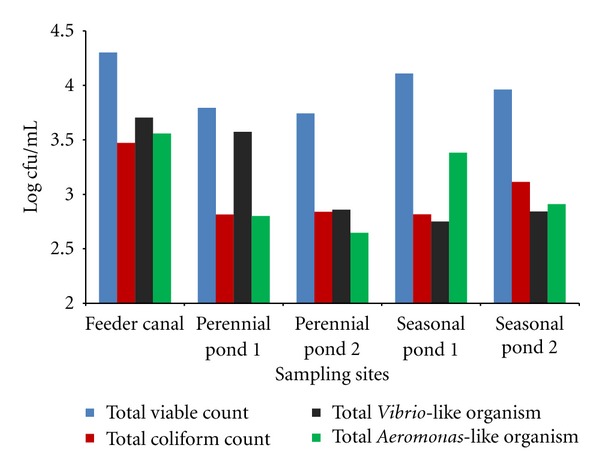
Mean value of bacteriological count of water from different sampling sites.

**Figure 2:**
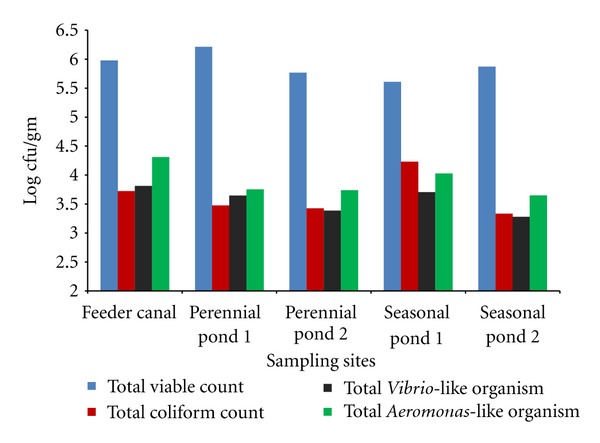
Mean value of bacteriological count of sediment from different sampling sites.

Figures 1 and 2 show the mean count of different groups of bacteria enumerated from water and sediment during the study period. The bacteriological analysis showed the THB load to be between 0.2 × 10^3^ to 9 × 10^4^ cfu/mL in water, and in the sediment, it was between 1.3 × 10^4^ to 1.79 × 10^7^ cfu/g. In water, TVLO count was found to be between 0–3.1 × 10^4^ cfu/mL, and TALO count was between 0.07 × 10^3^–1.43 × 10^4^ cfu/mL. TVLO and TALO of the sediment were 0–2.61 × 10^4^ and 0.8 × 10^3^–10.3 × 10^4^ cfu/g, respectively. In all the sampling stations, sediment was found to have higher microbial load than the water samples. TVC count was found to be generally higher in the feeder canal in contrast to both the perennial and seasonal ponds. In seasonal pond-2, TVLO were sometimes absent though the inoculum size (0.1 mL) was the same as in other ponds. The TC count of water and sediment were 0–2 × 10^4^ cfu/mL and 0–1.4 × 10^5^ cfu/g, respectively. Coliform count and Total *Vibrio*-like organisms count were also found to be higher in sediments than water. The same pattern was observed in the case of total *Aeromonas* count too. 

Table 3 shows the different genera of bacteria isolated from water, sediment, and shrimp in the months of September, 2009 and December, 2009. Genera of heterotrophic bacteria encountered were *Acinetobacter*, *Bacillus*, *Corynebacteria*, *Klebsiella*, *Kurthia*, *Listeria*, *Micrococcus*, *Moraxella*, *Staphylococcus*, *Alcaligenes*, *Cytophaga*, *Vibrio*. *Vibrio* sp, and *Aeromonas* sp. isolated from the feeder canal, perennial pond-1, perennial pond-2 are given in Table 4. Further species were characterization of Aeromonas and Vibrio isolates revealed the presence of *Aeromonas hydrophila* and various species of Vibrio such as *V. fluvialis*,* V. hollisae*,* V. vulnificus*,* V. furnissii*,* V. metschnikovii*, and* V. fischeri*.

### 3.3. WSSV Outbreak and Detection of WSSV by PCR

**Figure 3:**
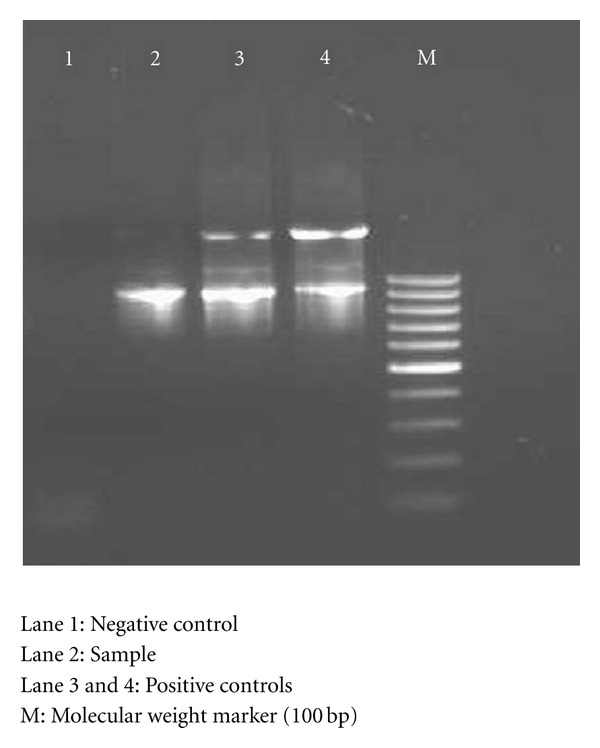
Results of PCR for WSSV from diseased shrimps from seasonal pond-1.

During the study period, WSSV outbreak occurred twice, once in the postmonsoon season (December, 2009) and once in the monsoon season (June–July, 2010), which was confirmed by PCR (Figure 3). Complete mortality was observed in seasonal pond-2 during the outbreak in June, 2010. There was no significant increase in the number of presumptive Vibrionaceae during the WSSV outbreak in December or in June, 2010. The result showed that the shrimps from both the seasonal ponds were under WSSV attack. The shrimp seasonal pond-1 also had parasitic *Zoothamnium* attack.

## 4. Discussion

Water and soil conditions influence shrimp production greatly that it is necessary to manage the pond properly for better results. Fluctuation of pH around the neutral value during culture period has been reported earlier, and it does not affect the growth of the shrimps, as the estuary is well buffered against serious changes in pH [21]. Results of the study revealed considerable dissolved oxygen stress in the feeder canal which is closely integrated to the seasonal ponds and the perennial ponds in the study region. Since the major seed supply to the farm is through natural water getting to the pond during high tide. These are normally retained in the pond and fattened to a marketable size. Over the years, the depth of the feeder canal has reduced considerably due to siltation and effluent from the nearby seafood preprocessing units and markets are drained into the feeder canal, which can considerably increase the nutrient load in the feeder canal and resulting in the reduction of dissolved oxygen. The seeds which gain entry into the pond might have subjected to dissolved oxygen stress and become susceptible to WSSV/opportunistic bacterial pathogens. Another important observation was periodical cyanobacterial bloom (identified as *Anabaenopsis* sp.—unpublished data) in the seasonal pond 2, which has limited water exchange. The dissolved oxygen in the pond was found to vary considerably during the day with hypoxic condition in the early morning (1.23–1.64 mg/L) and supersaturated dissolved oxygen conditions in the afternoon. This pond also shows alkaline pH to the tune of 9.7. WSSV affected shrimps were frequently encountered in these ponds. pH of the water samples were found to be within the acceptable range in the feeder canal, seasonal pond 1 and the perennial ponds, while that of the sediment from feeder canal occasionally showed acidic nature. Bottom soil quality is an important factor as the shrimps spend most of their time burrowing in the bottom and also ingest some of it [22]. Decreasing sediment pH or acidification of sediment (7.0–6.0) increases the hemolymph osmotic pressure of shrimps which is an indicator of variation in osmotic regulation. A significant decrease in hemolymph OP was seen as the water pH decreased from 7.0 to 6.5 [23]. 

Many studies have reported growth of *Penaeus monodon* in fresh water and low salinity as low as 2 ppt [22, 24–26]. Our observations have shown that the DO levels of the inlet canal is almost always very low and the canal is highly polluted by the wastes that are discharged into it from homes along the banks as well as seafood processing units in that area. Shrimp farmers have often expressed concerns over the polluted water surrounding their farms, as pollution by industrial, commercial, and urban contaminations [27] can slow growth, increase disease outbreaks, and accelerates the mortality rate of shrimps. Low levels of DO (<3.7 mg/L) when combined with high NH_3_-N (>0.5 mg/L) has shown to be harmful for P. *semisulcatus *[28]. L. E. Burnett and K. G. Burnett [29] suggested hypoxia results in a depression of the generalized innate immune response in *Paleomonetes pugio* and *Penaeus vannamei* on the basis of measurements of circulating hemocytes and survival of shrimp exposed to *Vibrio*. 

Guerrero-Galván et al. [30] have reported that during the rainy season, dissolved oxygen level tend to decrease, as the feeding rates and shrimp and phytoplankton biomass were increasing until harvest. A similar decrease in dissolved oxygen levels were seen during the monsoon season (diurnal changes in dissolved oxygen levels 1.24 mg/L at 7.00 a.m and around saturation in the afternoon) along with high phytoplankton biomass.

Presence of total ammonia nitrogen (TAN) can be attributed to the sludge that is deposited at the bottom of the ponds. In the ponds studied, there is no periodic removal of the settled sludge during the production cycle. During the past, the seasonal ponds were regularly used for paddy cultivation after a crop of shrimp, during which plowing and tilling operation were practiced before paddy cultivation. This was helpful in removing the sludge and utilization of it for growing paddy. Deepening of the perennial ponds was also prevented by farm owners, and the removed sludge and sediment were used for growing plants along the bunds of the farms. However, paddy cultivation and drying of perennial ponds are abandoned due to the unavailability of farm labour. Several studies have proved the contribution of settled sludge to ammonia production in shrimp growout ponds [31]. Yearsley et al. [32] have found that settled sludge that is not removed produces 44% more TAN than in tanks, where they are removed. These studies call for regular removal of sludge from the pond bottom to improve water quality as any increase in pH or temperature of the pond water can result in increased ammonia production which is lethal to shrimps. In closed systems, rapid accumulation of ammonia occurs and reduces growth even at low concentrations [21]. This could be the reason why the relatively huge perennial ponds were not affected though they had higher levels of TAN, whereas the smaller seasonal ponds 1 and 2 with low water exchange were both affected by WSSV and had higher TAN levels at that time.

High levels of TAN were recorded in the perennial ponds and the seasonal ponds. It was 2–180 times more than the safe level (0.01 mg/L NH_3_-N) calculated by Chin and Chen [33] for* P*. *monodon* larvae. The proportion of NH_3_ to NH_4_^+^ in water increases with increase in water temperature, pH and decrease in salinity. Unionized ammonia is toxic, as it has high lipid solubility and readily diffuses across cell membranes [34]. Even low levels can spell doom for shrimps if the pH increases as was the case seen in our study. Ammonia in water can suppress the immune system of *Littopenaeus vannamei *and increases mortality by* Vibrio alginolyticus* [35].

The nitrite levels recorded in our study were always lower than the safe limit (1.28 mg/L) recommended by Law [20]. This is in tune with a study in Taiwan between 1989 and 1990, where only few ponds (<0.5%) recorded nitrite concentrations higher than the safe level. These low levels are because they are intermediate products and unstable nature in the aquatic environment which limits their accumulation and consequently low concentrations [36].

Nitrate levels varied greatly (0.12–0.19 mg/L) and were found to be more than that recorded by Rao et al. [37]. The nitrate levels in their study were between 0.002–0.213 mg/L, whereas the lowest nitrate value (0.028 mg/L) itself was higher than that encountered in their study. The average nitrate levels recorded were also higher than what was recorded by [38] in the shrimp ponds of Thailand (0.08–0.16 mg/L). 

Bacterial count fluctuated during the study period, and there was no increase in the bacterial number, as the crop age increased. This trend is supported in a study by Burford et al. [39]. Total heterotrophic bacterial count in water was between 0.1 × 10^4^–9 × 10^4^ cfu/mL. They were lower compared to the levels recorded by Anand et al. [40] which was of the order of 10^6^–10^7^ cfu/mL. But the TVC counts of both water and sediment were found to be higher than that recorded by Janakiram et al. [41] in extensive and semi- intensive shrimp ponds in Andhra, Pradesh, India. 

 TVC levels in the sediment (1.3 × 10^4^–1.79 × 10^7^ cfu/g) were higher than that of the water. Water and sediment of the feeder canal showed relatively higher levels of TVC, TVLO, and TC count compared to the other ponds. Owing to the constant changes in the environment of a tidal estuary, it is difficult to study the influence of selected parameters on microbial population [42]. Janakiram et al. [41] also reported that a correlation between the physicochemical parameters and bacterial load could not be found. 

The high bacterial count in the sediment may be attributed to the presence of high organic matter and nutrients in the sediments than in the water column, as the THB count is dependent on the availability of nutrient sources and the sediment bacteria play a major role in the remineralisation of nutrients that accumulate at the pond bottom [43]. The heterotrophic bacteria oxidize the organic matter consuming oxygen and release carbon dioxide. Thus, the water quality in an aquaculture pond is greatly influenced by microbial degradation of organic matter. In all aquatic system including ponds, microbes are an integral part of the food web, thus directly influencing the productivity of the system. The heterotrophic bacterial community of an aquatic ecosystem is involved in the breakdown of complex organic compounds to simple molecules, hence providing an important food source [44, 45]. They are beneficial to shrimp ponds by their involvement in mineralization and regeneration of nutrients [46]. But the bacterial flora of the shrimp environment also has several facultative and opportunistic pathogens which turn virulent under poor environmental conditions leading to mass mortality of cultured shrimps [47, 48]. Species level characterization of Vibrio and Aeromonas revealed the presence of pathogenic strains. The prevalence of *Vibrio* sp. in the pond environment poses a threat to both humans and shrimps as *Vibrio *are pathogenic to humans and shrimps as well.* V. harveyi*,* V. vulnificus*,* V. parahemolyticus*,* V. anguillarum*, and* V. splendidus *are some of the *Vibrio* sp. reported as shrimp pathogens. They are common inhabitants of shrimp hatcheries, pond water, and sediment, and they turn pathogenic under poor environmental conditions [49]. In experimental studies, shrimp exposed to ammonium stress prior to challenge showed higher susceptibility to vibrios [35]. It has also been indicated that a primary WSSV infection may weaken shrimp, increasing their susceptibility to bacterial infections [50].

Higher coliform count in sediment than water observed in our study is supported by a similar work by Harish et al. [51]. Presence of fecal coliforms is an indicator of pollution, and it is a common factor in environments closer to human existence. Most of the bacteria encountered in a shrimp pond are opportunistic pathogens, as they are part of the natural ecosystem as well as the normal flora of the shrimp. We were able to isolate A. *hydrophila* from the water, sediment, and the shrimps. They are part of the normal flora of the shrimp environment and turn pathogenic under stressful conditions which are in most cases poor environmental and physiological parameters [52]. The pond water, diseased fish, frogs, and convalescent frogs may harbour motile aeromonads, which can cause septicemia under stress conditions such as low dissolved oxygen level, elevated temperature [53], elevated levels of ammonia, and carbon dioxide [54]. The ponds under study are extensive and traditional with other fish and crabs which would also play a role in disease outbreak, as they might harbor harmful bacteria.

Unlike terrestrial animals, which live in a fairly constant environment (stable oxygen and carbon dioxide content), shrimps live in an environment which changes often abruptly. Manifestation of diseases and stress are closely related, and stress is induced by sudden changes in the temperature, oxygen, salinity, and ammonium. Farmers often do not do anything to check the variability in the aquatic environment [55]. In a pond ecosystem, it is difficult to separate shrimps from other crustaceans like crabs and copepods, as they may be alternate hosts of shrimp diseases. Disease avoidance is the only way out for avoiding mortality and increasing production in these ponds. In South and South East Asia, shrimp farmers have some level of success in controlling WSSV by limiting water exchange which probably increases water temperature and prevents pathogens from entering the ponds [9]. Sengupta et al. [56] show how antibiotic resistant *Vibrio* sp. are transmitted through the feeder canal to growout ponds.

Though there are studies supporting that ammonia, nitrite, and hydrogen sulphide is toxic to shrimps, a single metabolite may not be held responsible for retarded growth and mortality of shrimps. Pai et al. [57] have reported that cumulative factors like sudden and steep decline in salinity, lowering of DO levels, increase in iron content in the hypolimnion following monsoon and subsequent increase in ammonia and hydrogen sulphide triggers the WSSV from latency to virulence, thus showing that more than one factor is always involved in disease outbreak. The amplification of viral loads and onset of disease can be induced by environmental or physiological stress or at ambient temperatures below 30°C.

Studies have shown the inability of WSSV to replicate in higher temperatures. Temperature reduction due to monsoon outpours in June and cold water conditions in December could be attributed to the outbreak of WSSV and species of the marine environment spread easily and are more devastating [58]. WSSV seems to be triggered or aggravated by changes in seawater quality including hardness, temperature, and dissolved oxygen [59]. A study by Jiang et al. [60] shows that both ammonia-N and WSSV decrease the plasma proteins and total hemocyte count of P. *japonicus* significantly which can lead to increased mortality. Plasma proteins play a major role in crustacean immune system and high level of ammonia-N in water is the main reason for decrease in plasma proteins. Due to high ambient ammonia-N, accumulation of haemolymph ammonia and urea occurs, leading to the catabolism of proteins to amino acids [61–63]. Fluctuations in temperature, pH, and salinity are important risk factors, which can lead to WSSV infection [64]. They have also found out that shrimps in less transparent deep ponds are more susceptible to WSSV infection than those in less transparent shallow ponds. 

Abrupt fluctuations in temperature and salinity due to heavy rain have been found to contribute to increase in viral loads in the shrimps and have caused 80% mortality in shrimps in a study in Mexico [65]. The same could be said of our study, as there was an outbreak of WSSV as soon as the monsoon started. Presence of the epicommensal protozoan, *Zoothamnium*, was a consistent occurrence among the WSSV-infected shrimp in the field outbreaks of India and Korea [66]. This is consistent with our observations too, as there was *Zoothamnium* infection in WSSV-positive P. *monodon* collected from the traditional ponds.

Along with low temperature, high pH and low dissolved oxygen caused by algal blooms (unpublished data) must have been a very stressful environment for the shrimps, aiding the rapid multiplication of WSSV and disease manifestation in the shrimps. It is very difficult to avoid diseases in an open-air culture system with close connection to the sea, by eliminating the causal agent or by using disease-free stocks. Rather, disease management based on providing conditions that will not help the development of the organism should be followed [9]. In our study period, there was no WSSV outbreak in perennial ponds, where feed input was either nil or minimal with almost stable dissolved oxygen and pH values throughout the culture period. In December, perennial pond-2 was treated with turmeric powder in an attempt to avoid disease outbreak which could have worked.

Bioaugmentation of the ponds with bacteria that can oxidize ammonia and nitrite could be used to improve water quality, as Rao et al. [37] have suggested that though these bacteria (involved in nitrogen and sulphur cycle) are present as natural flora of shrimp ponds, their numbers are low, particularly at the later part of the culture period. Survey conducted by Balasubramaniam et al. [67] in shrimp farms along the Chilka lagoon, Orissa, India, show that the production of zero-water exchange ponds has remained the same for years in that area, thus indicating the sustainability of the practice. Their production rate is also almost similar to the farms with regular water exchange. To replicate that sort of success in the seasonal farms along the Cochin backwaters, it is advised that farmers pay attention to water quality issues that plague them.

## Figures and Tables

**Table 1:** Water quality parameters of the feeder canal and the shrimp farms (mean value ± SD).

Parameter	Feeder canal	Perennial pond-1	Perennial pond-2	Seasonal pond-1	Seasonal pond-2
Temperature (°C)	29.95 ± 2.01 (27–36)*	30.75 ± 2.19 (28–36)	30.65 ± 2.23 (28–37)	30.45 ± 1.95 (28–36)	31.10 ± 2.26 (28–37)
Salinity (ppt)	11.45 ± 9.48 (0–30.00)	10.51 ± 7.92 (0–27.50)	10.41 ± 9.51 (0–30.80)	10.82 ± 9.61 (0–30.30)	10.63 ± 8.07 (2.97–29.00)
Dissolved oxygen (mg/L)	3.16 ± 1.59 (0.8–7.42)	5.23 ± 1.62 (2.0–7.01)	6.70 ± 1.79 (3.71–10.30)	6.62 ± 2.82 (2.88–13.00)	8.31 ± 2.80 (4.50–13.80)
pH	7.30 ± 0.2 (6.90–7.90)	7.60 ± 0.3 (7.27–8.40)	8.00 ± 0.5 (6.61–8.90)	7.68 ± 0.5 (6.86–8.60)	8.50 ± 0.8 (7.30–9.70)
Alkalinity (mgCaCO_3_/L)	62.40 ± 15.10 (24–84)	67.00 ± 15.23 (36–92)	62.60 ± 22.3 (16–92)	60.00 ± 18.17 (20–88)	68.20 ± 17.53 (20–88)
Total hardness (mgCaCO_3_/mL)	1684.2 ± 1230.192 (300–3820)	1695.8 ± 1066.57 (300–3600)	1647.0 ± 1276.354 (368–4200)	1695.0 ± 1328.06 (400–4400)	1698.0 ± 1172.346 (580–3900)
TAN (mg/L)	0.1958 ± 0.15 (0.00394–0.64600)	0.1758 ± 0.15 (0.05168–0.66348)	0.2498 ± 0.4 (0.00608–1.87112)	0.1647 ± 0.15 (0.01596–0.70680)	0.1287 ± 0.16 (0.00608–0.65892)
Nitrite (mg/L)	0.0149 ± 0.008 (0.00286–0.032186)	0.0102 ± 0.008 (0.00078–0.03059)	0.0214 ± 0.010 (0.00390–0.046284)	0.01504 ± 0.010 (0.000782–0.029792)	0.0185 ± 0.02 (0.00442–0.044688)
Nitrate (mg/L)	0.1748 ± 0.09 (0.057860–0.428164)	0.1205 ± 0.062 (0.02893–0.219868)	0.1930 ± 0.08 (0.046288–0.352946)	0.1397 ± 0.07 (0.052074–0.266156)	0.1379 ± 0.08 (0.005786–0.266156)
Phosphate (mg/L)	0.1840 ± 0.109 (0.05410–0.40254)	0.2728 ± 0.34 (0.06924–0.39168)	0.2928 ± 0.18 (0.12334–0.87425)	0.1998 ± 0.12 (0.03462–0.42631)	0.1658 ± 0.10 (0.01948–0.37437)

*Value in parentheses indicates range.

**Table 2:** Temperature and pH (Mean ± SD) of the sediment of feeder canal and the ponds.

Parameter	Feeder canal	Perennial pond-1	Perennial pond-2	Seasonal pond-1	Seasonal pond-2
Temperature (°C)	29.5 ± 1.88 (28–33)*	30.5 ± 2.02 (28–34)	30 ± 1.8 (28–33)	30.41 ± 1.5 (28–33)	30.5 ± 1.8 (28–33)
pH	7.6 ± 0.52 (6.11–7.8)	7.3 ± 0.31 (7-8)	7.3 ± 0.35 (7-8)	7.18 ± 0.52 (6.25–7.9)	7.36 ± 0.38 (6.9–8.1)

*Value in parentheses indicates range.

**Table 3:** Genera of heterotrophic bacteria identified from water, sediment, and shrimps in the feeder canal and shrimp farms.

Sl. no.	Bacterial genera identified from		
	Water	Sediment	Shrimp
1	*Acinetobacter*	*Alcaligenes *	*Alcaligenes*
2	*Bacillus*	*Bacillus *	*Bacillus*
3	*Corynebacteria *	*Corynebacteria*	*Corynebacteria*
4	*Klebsiella*	*Cytophaga*	*Listeria*
5	*Kurthia *	*Klebsiella*	*Micrococcus*
6	*Listeria *	*Listeria *	*Staphylococcus*
7	*Micrococcus*	*Micrococcus *	*Vibrio*
8	*Moraxella *	*Staphylococcus*	
9	*Staphylococcus *		

**Table 4:** Occurrence of *Aeromonas hydrophila* and *Vibrio* sp. from water, sediment, and shrimps in feeder canal and shrimp farms.

*Aeromonas/Vibrio* sp. identified	Sample
*Aeromonas* *hydrophila *	Water, sediment, and shrimp
*V*. *hollisae *	Water, sediment, and shrimp
*V*. *fluvialis *	Shrimp
*V*. *metschnikovii*, *V*. *fischeri *	Sediment
*V*. *furnissii*, *V*. *vulnificus *	Water
